# Complete Genome Sequence of Canine Papillomavirus Type 16

**DOI:** 10.1128/genomeA.00404-15

**Published:** 2015-05-07

**Authors:** Jennifer Luff, Michelle Mader, Monica Britton, Joseph Fass, Peter Rowland, Carolyn Orr, Richard Schlegel, Hang Yuan

**Affiliations:** a Department of Population Health and Pathobiology, College of Veterinary Medicine, North Carolina State University, Raleigh, North Carolina, USA; b Bioinformatics Core Facility, University of California at Davis, Davis, California, USA; c Histopath Consulting, Worcester, Maine, USA; d Animal Clinic of East Avenue, Rochester, New York, USA; e Department of Pathology, Georgetown University Medical School, Washington, DC, USA

## Abstract

Papillomaviruses are epitheliotropic, circular, double-stranded DNA viruses within the family *Papillomaviridae* that are associated with benign and malignant tumors in humans and animals. We report the complete genome sequence of canine papillomavirus type 16 identified within multiple pigmented cutaneous plaques and squamous cell carcinoma from an intact female Basenji dog.

## GENOME ANNOUNCEMENT

Papillomaviruses (PVs) are circular double-stranded DNA viruses associated with both benign and malignant epithelial proliferations (1). In dogs, cutaneous PV infections typically regress spontaneously; however, there are rare cases of malignant transformation to *in situ* or invasive squamous cell carcinoma (2–4). Identification and sequence analysis of specific PV types associated with a more malignant behavior is particularly valuable for studies on viral oncogenesis and the host immune response to PV infections (4–7).

This report describes the complete viral genome of a novel canine papillomavirus type, designated canine papillomavirus type 16 (CPV-16), identified from pigmented cutaneous plaques that progressed to squamous cell carcinoma in a female Basenji dog. Total viral DNA was isolated from both pigmented plaques and samples of squamous cell carcinoma by routine methods. PCR using degenerate consensus primers for the L1 gene was first used to amplify potential PV genome fragments. Sequencing results yielded a putatively novel PV DNA sequence. Samples of squamous cell carcinoma were the only fresh tissues available, and traditional methods used to sequence papillomaviruses proved unsuccessful. As an alternative, next-generation sequencing (NGS) using the Illumina, HiSeq 2500 platform was performed. The sequences generated from NGS were aligned to the *Canis familiaris* genome using BWA’s short-read aligner (8), and read pairs that aligned to the dog genome were set aside. The remaining reads were run through the PRICE assembler seeded with two approximately 400-bp segments of viral genome that were amplified and sequenced using degenerate primers (9). This resulted in a 4,920-bp fragment of the viral genome. The remaining viral genome sequence was obtained using viral DNA from a pigmented plaque, which generated a combination of overlapping amplicons using specific primers based upon the NGS sequencing results and additional degenerate primers. Vector NTI Advance 10 software (Invitrogen) was used to assemble the sequence contigs containing high-quality trace files. The results of the sequencing confirmed the presence of a novel CPV.

The complete viral genome for CPV-16 is 7,796 bp and encodes all of its open reading frames (ORFs) on the same coding strand of its circular double-stranded genome. CPV-16 has eight ORFs, including the six early (E) genes E1, E2, E4, E5, E6, and E7, and the two late (L) genes L1 and L2. The L1 gene is the most conserved gene within the genome and has been used for the identification and classification of new PVs. A new PV is recognized if the DNA sequence of the L1 ORF differs by more than 10% from all known PV types (1). The CPV-16 L1 gene is most closely related (70.5% homology) to CPV-9. This report will facilitate future investigations into the molecular pathogenesis and oncogenesis of CPV infections.

### Nucleotide sequence accession number.

The complete genome sequence of canine papillomavirus type 16 (CPV-16) is available in GenBank under the accession number NC_026640.
